# Isolated primary pericardial sarcomatoid mesothelioma: a case report

**DOI:** 10.3389/fonc.2026.1817459

**Published:** 2026-04-13

**Authors:** Pouya Nezafati, Ricardo Vilain, Taranpreet Singh

**Affiliations:** 1 Department of Cardiothoracic Surgery, John Hunter Hospital, Newcastle, NSW, Australia; 2 Department of Pathology, John Hunter Hospital, Newcastle, NSW, Australia

**Keywords:** case report, mesothelioma, pericardial mesothelioma, pericardiectomy, sarcomatoid mesothelioma

## Abstract

**Background:**

Primary pericardial mesothelioma is an exceedingly rare malignancy, often diagnosed late due to nonspecific clinical and radiologic findings. We report a case of isolated pericardial mesothelioma progressing from mesothelioma *in situ* to invasive sarcomatoid disease, presenting with recurrent effusions and constrictive pericarditis.

**Case presentation:**

A 58-year-old male with a remote history of Hodgkin’s lymphoma (previously treated with chemotherapy and mediastinal radiotherapy) presented with progressive dyspnoea, orthopnoea, and recurrent pericardial and left pleural effusions. Initial diagnostic video-assisted thoracoscopic surgery (VATS) with mini-thoracotomy revealed densely adherent pericardium, intra-pericardial clots, and 1.5L of blood-stained pleural fluid. Histology demonstrated a sparse population of surfcatypical mesothelial proliferation with methylthioadenosine phosphorylase (MTAP) loss and retained BRCA1-associated protein 1 (BAP1). There was no invasive growth and therefore, the appearance for concerning for mesothelioma *in situ*, at a minimum. Cytology of the pleural fluid was non-malignant. Due to progressive constrictive physiology, a total pericardiectomy via median sternotomy was performed with femoro-femoral cardiopulmonary bypass. Intraoperatively, the pericardium was excised in the standard fashion for total pericardiectomy, and cardiopulmonary bypass was required due to dense epicardial adhesions. Final histopathology confirmed sarcomatoid mesothelioma with high-grade spindle cell morphology, brisk mitotic activity, necrosis, and MTAP loss. Immunohistochemistry was positive for WT1, calretinin, CK5/6, and D2-40. The patient was referred for oncology follow-up and underwent PET-CT staging for systemic therapy planning.

**Conclusion:**

This case highlights the importance of early diagnostic suspicion in patients with prior mediastinal irradiation who present with recurrent pericardial effusions or evolving constrictive physiology. While pericardiectomy remains the standard of care for constriction regardless of etiology, the diagnosis of mesothelioma—particularly sarcomatoid subtype—warrants timely intervention. Early recognition of pre-invasive histological changes may offer a critical opportunity for curative resection before progression to aggressive disease.

## Introduction

Primary pericardial mesothelioma is a rare malignancy of mesothelial origin arising from the serosal lining of the pericardium. It accounts for a small fraction of primary cardiac tumors and an even smaller subset of all mesotheliomas, most of which are pleural. Because of its rarity and often indolent presentation, diagnosis is frequently delayed. Clinical manifestations include recurrent pericardial effusion, cardiac tamponade, or constrictive pericarditis (1).

Underdiagnosis remains a major issue, as symptoms are often attributed to viral or idiopathic pericarditis. Awareness among cardiologists and cardiothoracic surgeons is essential, especially for patients with recurrent pericardial disease or prior thoracic irradiation. Unlike pleural mesothelioma, asbestos exposure is less frequently implicated. Instead, prior mediastinal radiotherapy, chronic inflammation, and genetic susceptibility may play more prominent roles in tumorigenesis (2).

Recent advances in immunohistochemistry and molecular diagnostics have enabled earlier detection of mesothelial transformation. Lesions with methylthioadenosine phosphorylase (MTAP) or BRCA1-associated protein 1 (BAP1) loss in the absence of invasion are now classified as mesothelioma in situ—a potential precursor to invasive disease. Identifying such early changes in high-risk individuals may justify early surgical intervention (3).

## Case presentation

A 58-year-old man presented with progressive exertional dyspnea, orthopnea, anorexia, and weight loss. He denied chest pain and had no evidence of tamponade. His history included Hodgkin lymphoma treated with chemotherapy and mediastinal radiotherapy over 20 years earlier, splenectomy, and pacemaker implantation. There was no asbestos or other occupational exposure.

Transthoracic echocardiography showed preserved left ventricular function, biatrial enlargement, and a small pericardial effusion. Transesophageal echocardiography demonstrated a left atrial thrombus, septal bounce, and respiratory variation in mitral inflow—features consistent with evolving constrictive physiology. CT imaging revealed diffuse pericardial thickening and nodularity with a left pleural effusion. Serial pericardiocenteses yielded hemorrhagic, exudative fluid negative for malignancy.

A diagnostic video-assisted thoracoscopic surgery (VATS) with mini-thoracotomy was performed. Intraoperatively, 1.5 L of hemorrhagic pleural fluid was drained. The pericardium was thickened, nodular, and densely adherent, precluding a pericardial window. Pleural and pericardial biopsies were obtained.

Histopathology showed sparse atypical mesothelial proliferation with spindle morphology, MTAP loss, and retained BAP1—findings consistent with mesothelioma *in situ*. Immunohistochemistry was positive for calretinin, AE1/3, and CK5/6.

With radiologic progression and clinical decline, total pericardiectomy was undertaken via median sternotomy under femoro-femoral cardiopulmonary bypass due to dense adhesions. The pericardium appeared diffusely thickened and nodular and was excised completely.

**Figure 1:**
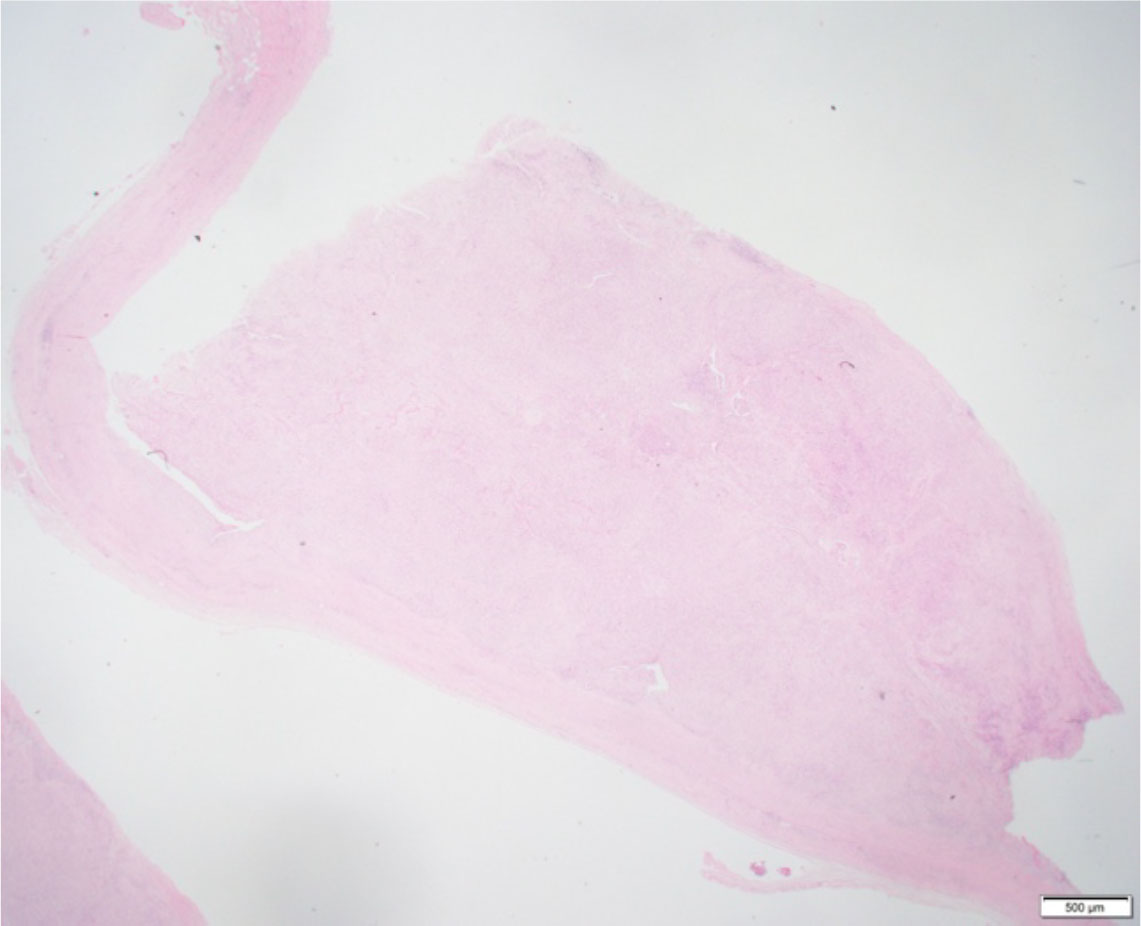
Low-power (×4) hematoxylin–eosin–stained section showing diffuse pericardial thickening and nodular infiltration by malignant mesothelial proliferation. The tumor involves the full pericardial thickness, replacing normal fibrous tissue and extending to the pericardial surface. This panoramic view highlights the infiltrative growth pattern characteristic of invasive sarcomatoid mesothelioma.

**Figure 2:**
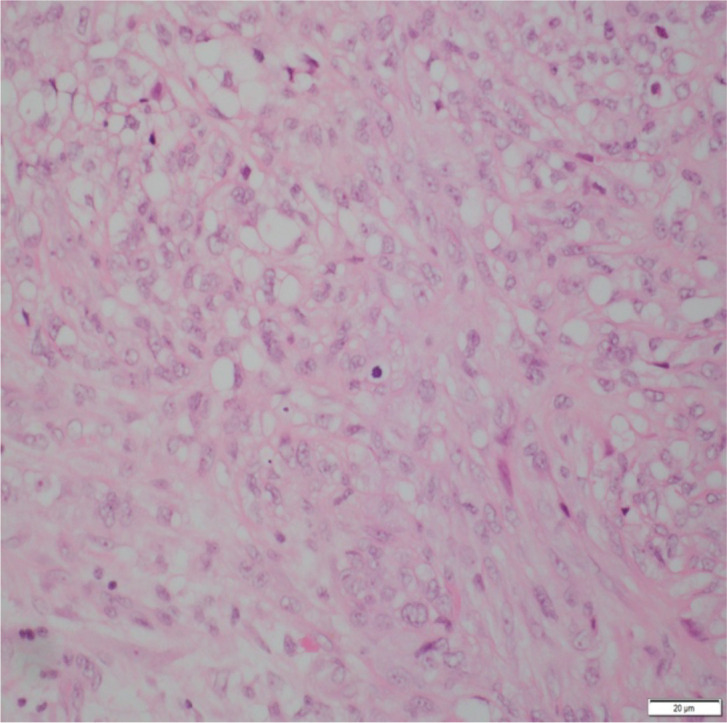
High-power hematoxylin–eosin section showing fascicles of malignant spindle cells with pleomorphic nuclei, coarse chromatin, and brisk mitotic activity, consistent with high-grade sarcomatoid mesothelioma. Focal necrosis and loss of pericardial architecture are evident, supporting an invasive phenotype.

**Figure 3:**
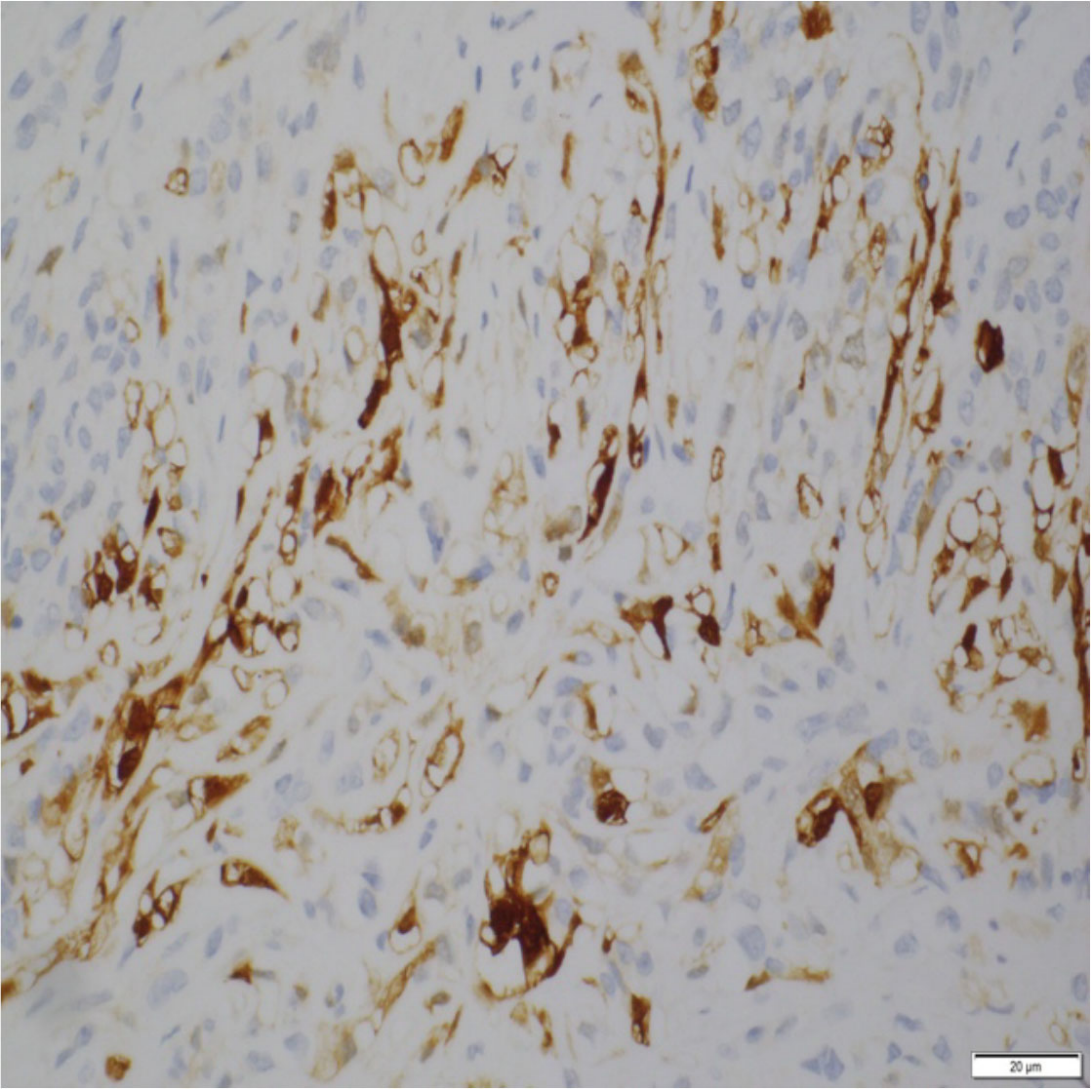
Immunohistochemical staining for calretinin showing strong cytoplasmic and nuclear positivity in spindle tumor cells, confirming mesothelial lineage. This pattern supports the diagnosis of sarcomatoid mesothelioma.

Low-power histology (**Figure 1**) demonstrated diffuse pericardial thickening and nodular infiltration by malignant spindle cells replacing fibrous tissue—diagnostic of invasive sarcomatoid mesothelioma. High-power sections (**Figure 2**) showed fascicles of pleomorphic spindle cells with brisk mitotic activity and necrosis. Immunostaining (**Figure 3**) demonstrated strong calretinin and WT1 positivity, with persistent MTAP loss, confirming sarcomatoid pericardial mesothelioma.

Postoperative recovery was uneventful. The patient was referred for oncologic evaluation and PET-CT for staging and systemic therapy planning.

## Discussion

Pericardial mesothelioma is one of the rarest and most difficult-to-diagnose cardiac malignancies. Its symptoms overlap with benign pericardial disease, and cytology from effusions is frequently non-diagnostic. In this case, suspicion was heightened by recurrent effusion, constrictive features, and a history of mediastinal irradiation—a recognized but uncommon risk factor (2).

Molecular markers such as MTAP and BAP1 loss have improved early detection. Recognition of mesothelioma *in situ* represents a potential window for curative intervention before progression. Although such findings are not definitively malignant, when accompanied by clinical deterioration, surgical resection should be strongly considered (3).

Sarcomatoid mesothelioma represents the least common but most aggressive histologic subtype, accounting for approximately 10% of mesothelioma cases. It is characterized by spindle-shaped tumor cells, high mitotic activity, and early invasive behavior, which contribute to significantly poorer prognosis and limited responsiveness to systemic therapy compared with epithelioid variants (4). In this patient, the evolution from mesothelioma *in situ* to overt sarcomatoid disease was demonstrated across serial specimens.

Sarcomatoid differentiation has also been described across a range of epithelial malignancies and may occur in tumors arising within previously irradiated tissues (5). Prior mediastinal radiotherapy is recognized as a risk factor for secondary malignancies, and long-term survivors of Hodgkin lymphoma treated with thoracic irradiation have an increased risk of developing aggressive secondary tumors after latency periods typically exceeding two decades (6). Although the relationship between radiation exposure and sarcomatoid differentiation itself remains incompletely understood, radiation-induced genomic instability has been proposed as a potential mechanism contributing to aggressive histologic phenotypes (7). In the present case, the development of sarcomatoid mesothelioma more than twenty years after mediastinal radiotherapy is consistent with the latency intervals described for radiation-associated secondary malignancies. In addition, a recent systematic review and meta-analysis evaluating the relationship between ionizing radiation exposure and mesothelioma risk reported a potential association between prior radiotherapy and subsequent development of mesothelioma, although the available evidence remains limited and does not establish a definitive causal relationship (8). These findings suggest that prior radiation exposure may represent a contributing factor in selected cases and should be considered when evaluating patients with a history of thoracic irradiation.

Management of primary pericardial mesothelioma remains challenging because of its rarity and the absence of standardized treatment protocols. Surgical resection in the form of total pericardiectomy remains the mainstay of treatment for patients presenting with constrictive physiology or recurrent pericardial effusion, providing both symptomatic relief and definitive histopathologic diagnosis. In cases with dense epicardial adhesions or extensive tumor involvement, cardiopulmonary bypass may be required to facilitate safe and controlled resection, as was necessary in our patient. Systemic treatment strategies for pericardial mesothelioma are largely extrapolated from the management of malignant pleural mesothelioma (9). Platinum-based chemotherapy combined with pemetrexed remains the most commonly employed first-line regimen; however, outcomes for sarcomatoid subtypes are generally poor due to intrinsic resistance to cytotoxic therapy.

More recently, immune checkpoint inhibitors targeting programmed death receptor pathways, including PD-1 and CTLA-4 inhibition, have demonstrated improved survival in pleural mesothelioma and may represent a potential therapeutic option for advanced pericardial disease, although evidence for their use in this rare entity remains limited (5, 10). Evaluation of PD-L1 expression and the tumor immune microenvironment, including tumor-infiltrating lymphocytes, may further refine patient selection for immunotherapy and guide future management strategies in mesothelioma.

Early diagnosis relies on heightened clinical vigilance. Patients with prior thoracic irradiation or persistent pericardial disease unresponsive to standard therapy should undergo early surgical biopsy. A multidisciplinary approach—combining cardiology, cardiothoracic surgery, oncology, and pathology—is essential for timely management.

## Conclusion

This case highlights the importance of maintaining clinical suspicion for malignant pericardial disease in patients presenting with recurrent pericardial effusions and a history of prior mediastinal irradiation. Sarcomatoid mesothelioma represents the least common but most aggressive histologic subtype of mesothelioma and is associated with poor response to systemic therapy. In the present case, the development of sarcomatoid pericardial mesothelioma more than two decades after mediastinal radiotherapy raises the possibility that prior radiation exposure may have contributed to tumorigenesis, although a definitive causal relationship cannot be established. Recognition of this potential association is important when evaluating patients with prior thoracic irradiation who present with unexplained pericardial pathology.

## Data Availability

The original contributions presented in the study are included in the article/supplementary material. Further inquiries can be directed to the corresponding author.
